# Reduced circulating sTWEAK levels are associated with metabolic syndrome in elderly individuals at high cardiovascular risk

**DOI:** 10.1186/1475-2840-13-51

**Published:** 2014-02-24

**Authors:** Andrés Díaz-López, Mònica Bulló, Matilde R Chacón, Ramón Estruch, Joan Vendrell, Javier Díez-Espino, Montserrat Fitó, Dolores Corella, Jordi Salas-Salvadó

**Affiliations:** 1 Human Nutrition Unit, Faculty of Medicine and Health Sciences, IISPV, Universitat Rovira i Virgili, C/ Sant Llorenç, 21, Reus, Tarragona 43201, Spain; 2 CIBERobn Physiopathology of Obesity and Nutrition, Institute of Health Carlos III, Madrid, Spain; 3 The Biomedical Research Centres Network for Diabetes and Associated Metabolic Disorders (CIBERDEM), University Hospital of Tarragona Joan XXIII, IISPV, Universitat Rovira i Virgili, Tarragona, Spain; 4 Department of Internal Medicine, Hospital Clinic, Institut d’Investigació Biomèdica August Pi i Sunyer (IDIBAPS), University of Barcelona, Barcelona, Spain; 5 Primary Health Care Centre of Tafalla, Tafalla, Spain; 6 Cardiovascular Risk and Nutrition Research Group (CARIN), Research Programme in Inflammatory and Cardiovascular Disorders (RICAD), IMIM (Hospital del Mar Medical Research Institute), Barcelona, Spain; 7 Department of Preventive Medicine and Public Health, School of Medicine, University of Valencia, Valencia, Spain

**Keywords:** sTWEAK, Metabolic syndrome, Cardiovascular risk, Biomarkers, Insulin resistance

## Abstract

**Background:**

The circulating soluble TNF-like weak inducer of apoptosis (sTWEAK) is a cytokine that modulates inflammatory and atherogenic reactions related to cardiometabolic risk. We investigated the association between sTWEAK levels and metabolic syndrome (MetS) and its components in older subjects at high cardiovascular risk.

**Methods:**

Cross-sectional analysis of 452 non-diabetic individuals (men and women aged 55–80 years) at high cardiovascular risk. MetS was defined by AHA/NHLBI and IDF criteria. Logistic regression analyses were used to estimate odds ratios (ORs) for MetS and its components by tertiles of serum sTWEAK concentrations measured by ELISA.

**Results:**

sTWEAK concentrations were lower in subjects with MetS than in those without. In gender- and age-adjusted analyses, subjects in the lowest sTWEAK tertile had higher ORs for overall MetS [1.71 (95% CI, 1.07-2.72)] and its components abdominal obesity [2.01 (1.15-3.52)], hyperglycemia [1.94 (1.20-3.11)], and hypertriglyceridemia [1.73 (1.05-2.82)] than those in the upper tertile. These associations persisted after controlling for family history of diabetes and premature coronary heart disease, lifestyle, kidney function and other MetS components. sTWEAK concentrations decreased as the number of MetS components increased. Individuals in the lowest vs the upper sTWEAK tertile had an increased risk of disclosing greater number of MetS features. Adjusted ORs for individuals with 2 vs ≤1, 3 vs ≤1, and ≥4 vs ≤ 1 MetS components were 2.60 (1.09-6.22), 2.83 (1.16-6.87) and 6.39 (2.42-16.85), respectively.

**Conclusion:**

In older subjects at high cardiovascular risk, reduced sTWEAK levels are associated with MetS: abdominal obesity, hypertriglyceridemia and hyperglycemia are the main contributors to this association.

## Background

The TNF-like weak inducer of apoptosis (TWEAK) is a molecule of the TNF superfamily [1,2]. It is initially expressed as a type-II trans-membrane protein (mTWEAK), but a smaller biologically active form can also be shed into the circulation as a soluble form (sTWEAK). TWEAK is ubiquitously expressed in a variety of organs, tissues and cell types, with high levels in heart, brain, pancreas, skeletal muscle and adipose tissues, and lower levels in liver and kidney. The binding of sTWEAK to its receptor, the fibroblast growth factor-inducible 14 (Fn14), mediates multiple biological effects, including proinflammatory cytokine induction, angiogenesis, and the regulation of cell survival, proliferation and death [3,4].

In recent years, cross-sectional studies investigating the associations between sTWEAK and diseases and/or metabolic disorders have been widely reported. In this context, Blanco-Colio et al. reported lower levels of sTWEAK in patients with atherosclerosis and a negative correlation between circulating TWEAK levels and the intima/media thickness in asymptomatic subjects [5]. Similarly, circulating sTWEAK levels have been found to be reduced in various cardiovascular and metabolic disorders [6-11]. Decreased sTWEAK levels have also been related to a higher risk of endothelial dysfunction in subjects with chronic kidney disease [12], and to long-term cardiovascular mortality in patients with peripheral artery disease (PAD) [13]. And circulating sTWEAK concentrations have been negatively correlated with such other laboratory and clinical parameters as fasting plasma glucose levels, insulin resistance assessed by homeostasis model assessment of insulin resistance (HOMA-IR) and systolic or diastolic blood pressure in patients with type 2 diabetes (T2D) and in chronic kidney disease [10,14]. Finally, our group has demonstrated that patients with type 1 diabetes (T1D) had lower sTWEAK levels than healthy subjects [15], and a negative association between sTWEAK levels, glucose metabolism-related parameters and T2D incidence has been reported [16].

To date, the relationship between circulating sTWEAK levels and Metabolic Syndrome (MetS) has not been explored. Based on recent researches, we hypothesized that low serum sTWEAK levels are associated with MetS, a common metabolic disorder, resulting from abdominal obesity and insulin resistance [17]. Thus, the present study aimed to explore whether circulating sTWEAK concentrations are associated with MetS and its individual components in elderly subjects at high cardiovascular risk.

## Methods

### Study design and participants

The present cross-sectional study was carried out on 452 Caucasian individuals at high risk for coronary heart disease (CHD), recruited by their medical practitioners from three Spanish centers (Reus, Barcelona and Pamplona) within the framework of the PREDIMED study [18], which were randomly selected from those without diabetes at baseline. The participants studied were community-dwelling men and women aged 55–80 years. The exclusion criteria included: prior cardiovascular disease (CVD) (including coronary heart disease [angina, myocardial infarction, coronary revascularization procedures or existence of abnormal Q waves in the electrocardiogram], stroke [either ischemic or hemorrhagic, including transient ischemic attacks], or clinical peripheral artery disease with symptoms of intermittent claudication), prevalent diabetes or treatment with insulin or oral hypoglycemic drugs, immunodeficiency or HIV-positive status, illegal drug use or chronic alcoholism, and/or body mass index (BMI) >40 kg/m^2^. All participants had at least three of the following cardiovascular risk factors: smoking, hypertension (systolic blood pressure ≥140 mmHg or diastolic blood pressure ≥90 mmHg or under antihypertensive medication), high low-density lipoprotein (LDL)-cholesterol (≥160 mg/dl), low high-density lipoprotein (HDL)-cholesterol (≤40 mg/dl independently of lipid-lowering therapy), overweight/obesity (BMI ≥25 kg/m^2^), or family history of early-onset CHD.

The study was conducted in agreement with the Declaration of Helsinki. The study protocol was approved by the institutional review boards of the three centers involved, and all subjects agreed to participate in the study and gave their informed consent.

### Clinical and anthropometric information

Medical information was collected on subjects’ medical record of a 47-item questionnaire about education, lifestyle, history of illnesses and medication use. We also administered a semiquantitative 137-item validated food frequency questionnaire [19] and a validated Spanish version of the Minnesota Leisure Time Physical Activity Questionnaire to assess physical activity [20]. In addition, anthropometric variables such as weight, height and waist circumference (midway between the lower rib and the superior border of the iliac crest) and blood pressure measured in triplicate using a validated semi-automatic oscillometer (Omron HEM-705CP, Hoofddorp, Netherlands) were determined by trained staff [21].

### Laboratory measurements

Blood samples were collected from all participants after an overnight fast. The aliquots of serum and EDTA plasma were immediately processed at 4°C, coded, and shipped to a central laboratory in a portable cooler, and stored at -80°C until analysis. The time between blood sampling and freezing was less than one hour. Plasma levels of fasting glucose and serum levels of total cholesterol, HDL-cholesterol and triglycerides were measured by routine laboratory tests using standard enzymatic methods. LDL-cholesterol concentrations were calculated using Friedewald’s equation. Serum and urinary creatinine, and urinary albumin concentrations were determined by the modified Jaffé colorimetric method and by the Bromcresol green albumin (BCG) method, respectively.

The urinary albumin (mg/L)-to-creatinine (mg/dL) ratio (ACR) was calculated and is reported in milligrams per gram. On the basis of serum creatinine concentration measurements, the glomerular filtration rate was estimated (eGFR) by the CKD-EPI (Chronic Kidney Disease Epidemiology Collaboration) equation for Caucasians [22] as a measure of kidney function. sTWEAK concentrations were determined in duplicate, in serum samples that had never suffered any previous thaw cycle, by ELISA using the commercially available, human TWEAK/TNFSF12 kit #DY1090 (R&D Systems Europe, Abingdon, Oxon, UK). The intra- and inter-assay coefficients of variation were 2.5% and 7.0%, respectively.

### Definition of metabolic syndrome

The definition of MetS that we used was the new one by the International Diabetes Federation (IDF) and the American Heart Association/National Heart, Lung, and Blood Institute (AHA/NHLBI), which has harmonized previous definitions [23]. According to this definition, a diagnosis of MetS requires the presence of at least three of the following five components or risk factors: (1) abdominal obesity measured by waist circumference according to different ethnic groups by gender; (2) hypertension (systolic and/or diastolic blood pressures ≥130/85 mmHg or antihypertensive drug treatment); (3) low HDL-cholesterol levels [fasting serum HDL-cholesterol levels <40 mg/dL (1.03 mmol/L) in men and <50 mg/dL (1.3 mmol/L) in women or drug treatment for reduced HDL-C; (4) hypertriglyceridemia [fasting serum triglycerides ≥150 mg/dL (1.7 mmol/L) or taking triglyceride-lowering medication]; and (5) hyperglycemia [fasting plasma glucose level ≥100 mg/dL (≥5.5 mmol/L) or drug treatment for elevated glucose levels].

### Statistical analysis

Analyses were performed using the SPSS software version 19.0 (SPSS Inc, Chicago, IL). General characteristics of the study population are presented by MetS status and were compared using independent *t*-tests for continuous variables and Pearson χ^2^ tests for dichotomous variables. The distribution of variables was tested using the Kolmogorov–Smirnov tests. Skewed variables (sTWEAK, triglyceride and plasma fasting glucose levels) were log_e_-transformed prior to statistical analyses to approximate a normal distribution. All the data are presented either as means ± standard deviation (SD) for symmetric continuous variables, median and inter quartile range for skewed continuous variables, or numbers and percentages for dichotomous variables. Age- and gender-adjusted partial Pearson correlation analysis was used to study the relationships between serum sTWEAK concentration and each of the MetS components (all as continuous variables).

Binary logistic regression analysis was used to estimate the odds ratios (ORs) and 95% confidence intervals (CI) for the prevalence of MetS, its components separately, and the MetS score [sum of MetS components for each individual (≤1 to ≥4)] by sTWEAK tertiles. The upper sTWEAK tertile was considered as the reference category. For the regression analyses several models were fitted, controlling for the following potential confounders: Model 1 was adjusted for gender and age. Model 2 was additionally adjusted for family history of diabetes and premature CHD (yes/no), educational level (primary education vs secondary/higher education), alcohol intake (g/d), smoking (current/not current), physical activity [sedentary (≤200 METs-min/d) vs active (>200 METs-min/d)] and eGFR. A third model was also fitted to assess the associations between tertiles of sTWEAK concentrations and each individual component of the MetS. This model was additionally adjusted for the above-mentioned model 2 and for the other components of MetS as dichotomized variables (model 3). Effect modification by gender was evaluated and tested by adding the product term of serum sTWEAK concentration*gender to the univariate model. The models were not stratified by gender because no significant interactions between sTWEAK and gender on MetS or its components were detected (all *P*-values >0.05). Linear trend tests were calculated using the median serum sTWEAK concentrations of each category, which were then introduced into the models as a continuous variable. All statistical tests were two-tailed and the significance level was set at P <0.05.

## Results

Table 1 presents the general characteristics of the study populations stratified by MetS status. In all the individuals (n = 452; mean age, 66 ± 6 year) – 214 men and 238 women – the prevalence of MetS was 51.8% (234/452). Those with MetS exhibited significantly higher mean BMI, waist circumference, and systolic and/or diastolic blood pressure than those without. They also had significantly higher levels of serum triglycerides and plasma glucose. In contrast, individuals with MetS had significantly lower HDL-cholesterol levels and serum sTWEAK concentrations than those subjects without MetS (Table 1).The age- and gender-adjusted partial correlation analysis in the whole population showed that sTWEAK concentrations were negatively related to waist circumference (r = -0.13, p =0.005), fasting plasma glucose (r = -0.16, p <0.001), and serum triglyceride levels (r = -0.10, p =0.03). No relationship between sTWEAK concentrations and HDL-cholesterol levels, or systolic and/or diastolic blood pressure was found (data not shown). We also used the same partial correlation analysis to assess the relationship between sTWEAK concentrations and such markers of kidney function as urinary ACR (ACR data was available for only 246 participants) and eGFR. No relationship was found between sTWEAK and ACR (r = 0.05; P =0.42). However, a weak inverse relationship was observed between sTWEAK and eGFR (r = -0.14; P = 0.003). Even so, we included this variable in the model as a confounder.

**Table 1:** General characteristics of the study population stratified for the presence and absence of MetS

**Baseline variable**	**MetS**	**Non-MetS**	P value*
**n**	234	218	
**Age, years**	66 ± 6	67 ± 6	0.30
**Men, % (n)**	47 (103)	47 (111)	1.00
**BMI, kg/m**^**2**^	30.6 ± 2.9	29.2 ± 3.0	<0.001
**Overweight/obesity (BMI ≥25 kg/m**^**2**^**), % (n)**	97 (228)	94 (206)	0.14
**Waist circumference, cm**	102.6 ± 8.8	96.5 ± 9.8	<0.001
**Smoking habits**			
**Current smoker, % (n)**	23 (54)	22 (48)	0.82
**Non-smoker, % (n)**	77 (180)	78 (170)	
**Physical activity, METS-min/d**	255.2 ± 205.8	289.0 ± 265.0	0.13
**Educational level**				
**Primary education, % (n)**	69 (160)	71 (154)	0.61	
**Secondary or higher education, % (n)**	31 (74)	29 (64)		
**Family history of diabetes, % (n)**	31 (73)	25 (55)	0.17	
**Family history of premature CHD, % (n)**	16 (38)	18 (41)	0.49	
**Diastolic blood pressure, mmHg**	88.6 ± 10.4	84.2 ± 10.0	<0.001	
**Systolic blood pressure, mmHg**	155.5 ± 20.5	149.0 ± 19.2	<0.001	
**LDL-cholesterol, mg/dL**	137.8 ± 32.2	140.6 ± 31.8	0.35	
**HDL-cholesterol, mg/dL**	50.8 ± 12.0	59.4 ± 14.0	<0.001	
**Triglyceride, mg/dL**^**†**^	159.0 (116.0, 208.0)	107.0 (82.0, 130.0)	<0.001	
**Fasting glucose, mg/dL**^**†**^	105.3 (97.0, 117.6)	91.0 (86.0, 97.0)	<0.001	
**sTWEAK, pg/mL**^**†**^	543.0 (367.3, 751.2)	595.6 (418.6, 844.5)	0.014	
**Urinary ACR (mg/g)**	18.1 ± 31.9	12.7 ± 30.3	0.17	
**eGFR (ml/min/1.73 m**^**2**^**)**	74.0 ± 14.9	75.5 ± 15.3	0.29	
**Metabolic syndrome components**				
**Abdominal obesity, % (n)**	88 (207)	53 (115)	<0.001	
**Hyperglycemia, % (n)**	69 (161)	10 (22)	<0.001	
**Hypertriglyceridemia, % (n)**	60 (141)	6 (14)	<0.001	
**Low HDL-cholesterol level, % (n)**	37 (88)	5 (12)	<0.001	
**Hypertension, % (n)**	99 (233)	94 (206)	0.001	

Data expressed as mean ± SD, or % (number). *Abbreviations:MetS* Metabolic Syndrome, *BMI* body mass index, *METs* Metabolic Equivalents, *CHD* coronary heart disease, *LDL* low-density lipoprotein, *HDL* high-density lipoprotein, *sTWEAK* soluble TNF-like weak inducer of apoptosis, *ACR* Albumin–creatinine ratio (ACR data was available only for 246 participants; n = 129 in MetS group and n = 117 in Non-MetS group), *eGFR* estimated glomerular filtration rate. ^**†**^Values expressed as median with inter quartile range. *P value for comparisons between two groups was tested by χ2 test for categorical variables or Student’s t-test for continuous variables.

Binary logistic regression analyses are reported in Tables 2, 3 and 4. The prevalence of MetS according to sTWEAK levels in the lowest, middle, and highest tertile decreased from 59.3 to 49.7 and 46.4%, respectively. In the crude analysis, individuals in the lower sTWEAK tertile had a significant higher risk of MetS than subjects in the upper tertile [OR, 1.69 (95% CI, 1.06 - 2.66); P for trend = 0.024]. After adjustments for relevant confounders (such as gender, age, family history of diabetes and premature CHD, education, smoking, alcohol intake, physical activity and eGFR) the magnitude of these associations did not appreciably change [1.85 (1.15 - 3.00); P for trend = 0.011]. A similar and significant inverse association between sTWEAK and prevalence of MetS was observed when the sTWEAK was analyzed as a continuous variable [1.49 (1.13 - 1.98) per unit decrease in estimated log sTWEAK] (Table 2).

**Table 2:** **Odds ratio and 95**% **CI for MetS according to tertiles of serum sTWEAK concentrations**

		**Tertiles of sTWEAK concentrations**			
	**Continuous**^**‡**^	**1st tertile**	**2nd tertile**	**3rd tertile**	P value for trend*
		**<453.1 ng/mL**	**453.1 to 706.4 < ng/mL**	**≥706.4 ng/mL**	
**No. of MetS/No. of non-MetS**	**234/218**	**89/61**	**75/76**	**70/81**	
Crude model	1.39 (1.06 - 1.82)	1.69 (1.06 - 2.66)	1.14 (0.73 - 1.79)	1.00 [Ref.]	0.024
Model 1	1.40 (1.07 - 1.84)	1.71 (1.07 - 2.72)	1.15 (0.72 - 1.82)	1.00 [Ref.]	0.022
Model 2	1.49 (1.13 - 1.98)	1.85 (1.15 - 3.00)	1.21 (0.75 - 1.94)	1.00 [Ref.]	0.011

Results expressed as odds ratios (95% CI) for MetS from logistic regression analysis. *Abbreviations*: *MetS* Metabolic Syndrome, *sTWEAK* soluble TNF-like weak inducer of apoptosis.

Crude model: unadjusted; Model 1: Adjusted for gender and age; Model 2: Additionally adjusted for family history of diabetes and premature CHD (yes/no), educational level (primary education *vs* secondary/higher education), alcohol intake (g alcohol/d), smoking (current/not-current), physical activity (sedentary vs active) and eGFR. ^‡^Per unit decrease in log sTWEAK. *Linear trend tests were calculated using the median serum sTWEAK concentrations of each category and using them as a new continuous variable in the models.

**Table 3:** **Odds ratio and 95**% **CI for the individual components of the MetS according to tertiles of serum sTWEAK concentrations**

	**Tertiles of sTWEAK concentrations**			
**Effects on components of the MetS**	**1st tertile**	**2nd tertile**	**3rd tertile**	P value for trend*
	**<453.1 ng/mL**	**453.1 to <706.4 ng/mL**	**≥706.4 ng/mL**	
**Abdominal obesity**				
No. abdominal obesity/No. non abdominal obesity	120/30	94/57	108/43	
Crude model	1.60 (0.93 - 2.71)	0.65 (0.40 - 1.06)	1.00 [Ref.]	0.091
Model 1	2.01 (1.15 - 3.52)	0.86 (0.51 - 1.43)	1.00 [Ref.]	0.012
Model 2	2.03 (1.14 - 3.60)	0.88 (0.52 - 1.47)	1.00 [Ref.]	0.013
Model 3	1.85 (1.03 - 3.33)	0.87 (0.51 - 1.47)	1.00 [Ref.]	0.036
**Hyperglycemia**				
No. hyperglycemia/No. non hyperglycemia	74/76	60/91	49/102	
Crude model	2.03 (1.27 - 3.23)	1.37 (0.86 - 2.19)	1.00 [Ref.]	0.003
Model 1	1.94 (1.20 - 3.11)	1.29 (0.80 - 2.09)	1.00 [Ref.]	0.006
Model 2	1.95 (1.20 - 3.19)	1.37 (0.84 - 2.24)	1.00 [Ref.]	0.007
Model 3	1.71 (1.04 - 2.83)	1.37 (0.82 - 2.27)	1.00 [Ref.]	0.037
**Hypertriglyceridemia**				
No. hypertriglyceridemia/No. nonhypertriglyceridemia	60/90	54/97	41/110	
Crude model	1.79 (1.10 - 2.90)	1.49 (0.92 - 2.46)	1.00 [Ref.]	0.021
Model 1	1.73 (1.05 - 2.82)	1.40 (0.85 - 2.31)	1.00 [Ref.]	0.031
Model 2	1.97 (1.18 - 3.30)	1.44 (0.86 - 2.42)	1.00 [Ref.]	0.010
Model 3	1.68 (1.02 - 2.79)	1.36 (0.82 - 2.26)	1.00 [Ref.]	0.041
**Low HDL-cholesterol**				
No. low HDL-cholesterol/No. non low HDL-cholesterol	31/119	31/120	38/113	
Crude model	0.77 (0.45 - 1.33)	0.76 (0.44 - 1.31)	1.00 [Ref.]	0.355
Model 1	0.81 (0.47 - 1.41)	0.81 (0.47 - 1.42)	1.00 [Ref.]	0.478
Model 2	0.86 (0.48 - 1.52)	0.82 (0.46 - 1.46)	1.00 [Ref.]	0.614
Model 3	0.67 (0.36 - 1.24)	0.71 (0.39 - 1.29)	1.00 [Ref.]	0.207
**Hypertension**				
No. hypertension/No. non hypertension	148/2	143/8	148/3	
Crude model	1.50 (0.25 - 9.10)	0.36 (0.09 - 1.39)	1.00 [Ref.]	0.697
Model 1	1.51 (0.25 - 9.30)	0.37 (0.09 - 1.47)	1.00 [Ref.]	0.653
Model 2	1.67 (0.25 - 11.26)	0.33 (0.08 - 1.36)	1.00 [Ref.]	0.619
Model 3	1.22 (0.18 - 8.42)	0.33 (0.07 - 1.41)	1.00 [Ref.]	0.929

Results expressed as odds ratios (95% CI) for MetS components from logistic regression analysis. *Abbreviations*: *MetS* Metabolic Syndrome, *sTWEAK* soluble TNF-like weak inducer of apoptosis.

Crude model: unadjusted; Model 1: Adjusted for gender and age; Model 2: Additionally adjusted for family history of diabetes and premature CHD (yes/no), educational level (primary education vs secondary/higher education), alcohol intake (g alcohol/d), smoking (current/not-current), physical activity (sedentary vs active) and eGFR; Model 3: Additionally adjusted for the presence of abdominal obesity (except for abdominal obesity), hyperglycemia (except for hyperglycemia), hypertriglyceridemia (except for hypertriglyceridemia), low HDL-cholesterol (except for low HDL-cholesterol), hypertension (except for hypertension). *Linear trend tests were calculated using the median serum sTWEAK concentrations of each category and using them as a new continuous variable in the models.

**Table 4:** **Odds ratio and 95**% **CI for MetS score (number of MetS components) according to tertiles of serum sTWEAK concentrations**

	**Tertiles of sTWEAK concentrations**		
**MetS score**	**1st tertile**	**2nd tertile**	**3rd tertile**
	**<453.1 ng/mL**	**453.1 to <706.4 ng/mL**	**≥706.4 ng/mL**
Crude model:			
2 *vs* ≤1	2.15 (0.96 - 4.79)	0.96 (0.49 - 1.88)	1.00 [Ref.]
3 *vs* ≤1	2.42 (1.06 - 5.51)	1.11 (0.56 - 2.22)	1.00 [Ref.]
≥4 *vs* ≤1	3.88 (1.66 - 9.06)	1.11 (0.52 - 2.36)	1.00 [Ref.]
Model 1:			
2 *vs* ≤1	2.84 (1.22 - 6.62)	1.26 (0.62 - 2.58)	1.00 [Ref.]
3 *vs* ≤1	2.90 (1.23 - 6.81)	1.39 (0.66 - 2.90)	1.00 [Ref.]
≥4 *vs* ≤1	4.50 (1.87 - 10.82)	1.33 (0.61 - 2.93)	1.00 [Ref.]
Model 2:			
2 *vs* ≤1	2.60 (1.09 - 6.22)	1.24 (0.60 - 2.58)	1.00 [Ref.]
3 *vs* ≤1	2.83 (1.16 - 6.87)	1.45 (0.67 - 3.01)	1.00 [Ref.]
≥4 *vs* ≤1	6.39 (2.42 - 16.85)	1.92 (0.80 - 4.61)	1.00 [Ref.]

Results expressed as odds ratios (95% CI) for the number of MetS components from logistic regression analysis. Sample size of each group of MetS components score is as follows: ≤1, n = 62; 2, n = 156; 3, n = 130; ≥4, n = 104. *Abbreviations*: *MetS* Metabolic Syndrome, *sTWEAK* soluble TNF-like weak inducer of apoptosis.

Crude model: unadjusted; Model 1: Adjusted for gender and age: Model 2: Additionally adjusted for family history of diabetes and premature CHD (yes/no), educational level (primary education vs secondary/higher education), alcohol intake (g alcohol/d), smoking (current/not-current), physical activity (sedentary vs active) and eGFR.

Similarly, after adjusting for model 2 mentioned above, compared to the subjects in the upper sTWEAK tertile, those in the lower tertile had an increased risk of the abdominal obesity, hyperglycemia, and hypertriglyceridemia components of the MetS [2.03 (1.14 - 3.60), P for trend 0.013; 1.95 (1.20- 3.19), P for trend 0.007; 1.97 (1.18-3.30), P for trend 0.041, respectively]. These associations were slightly attenuated but remained significant after additional adjustment for the others MetS components (model 3). Nevertheless, sTWEAK was not associated with the prevalence of such components of the MetS as low HDL-cholesterol and hypertension (Table 3).

A total of 13.7% of the total study population had one or no components of the MetS, 34.5% had two, 28.8% had three, and 23.0% had four or more. Serum sTWEAK concentrations decreased when the number of MetS components (MetS score) was increased. The median sTWEAK concentrations in the following categories: ≤1, 2, 3, and ≥4 MetS components were 615.6, 587.5, 555.7, and 522.1 pg/mL, respectively (P for trend = 0.001). Table 4 shows the ORs for the MetS score associated with the presence of two, three, and four or more MetS components compared with the presence of one or no components, according to sTWEAK tertiles. In the full model (model 2), individuals in the lowest *vs* the upper sTWEAK tertile had an increased risk of having a greater number of MetS components.

## Discussion

In this cross-sectional study conducted on community-dwelling elderly subjects at high cardiovascular risk, we report for the first time that low sTWEAK concentrations were associated with increased risk of MetS, independent of several confounders. Abdominal obesity, hyperglycemia and hypertriglyceridemia were the main contributors to this association. These findings add new knowledge to the current scientific literature, and suggest that sTWEAK may have a role in metabolic disorders such as MetS.

Overall, almost all the available studies have confirmed that patients with CVD or CVD-related diseases have decreased sTWEAK concentrations. In previous studies, a reduced concentration of sTWEAK has been described in patients with such pathological conditions as chronic heart failure [6], coronary and peripheral arterial disease [7-9], hypertension [11], T1D and T2D, and/or end-stage renal failure [10,15,16]. These data are in agreement with the findings of our study, which indicated a link between low sTWEAK concentrations and MetS, a cluster of metabolic disorders which predispose to the development of T2D and atherosclerosis, and increase the risk of CVD [24]. In line with this, several authors have suggested that reduced levels of sTWEAK might serve as a novel biomarker of atherosclerosis [5,10]. In fact, it has recently been reported that low sTWEAK levels were associated with long-term cardiovascular mortality in symptomatic PAD patients [13], and could be considered as a predictor of future cardiovascular outcomes in non-dialysis CKD patients [25]. It is also important to mention that in our study as the number of MetS components increased, the sTWEAK values gradually decreased. In keeping with these observations, we found that individuals with lower sTWEAK levels were more likely to have more MetS components. Thus, the consideration of sTWEAK as an indicative biomarker of risk factors for CVD is reinforced by our findings in MetS patients. Although our data support the possible existence of etiological associations, the biological mechanisms through which low sTWEAK levels were related to MetS remain speculative. Obesity-induced inflammation might be one of the mechanisms explaining the observed association in our study.

Vendrell et al. [26] reported that TWEAK and Fn14 gene expression were higher in the adipose tissue of severely obese patients than in controls, and that inflammatory stimuli in vitro induced by lipopolysaccharide and TNFα up-regulated TWEAK in THP-1 macrophages and Fn14 expression in SGBS adipocyte cells, respectively [26]. Despite the moderate inflammatory capacity of the sTWEAK cytokine over the adipocyte, a competitive interfering activity with TNFα signalling in the adipocyte has been described [27,28]. An imbalance of sTWEAK forms (membrane bound mTWEAK and soluble sTWEAK) in the obese state may help potentiate the inflammatory effect of TNFα over the adipocyte.

Our finding that lower sTWEAK concentrations were related with abdominal obesity agrees with the data obtained by Maymó-Masip et al. [27], which show that sTWEAK levels in severely obese patients are lower than in controls, and that their concentration increases after weight loss. This may suggest that sTWEAK plays an anti-inflammatory role in this setting, although additional in vivo and in vitro studies are required. Moreover, in the case of severely obese individuals they found that the main determinant of the sTWEAK circulating levels was BMI, in an inverse-dependent manner [27].

There is no clear explanation for the mechanisms leading to lower levels of sTWEAK in individuals with MetS. The rationale that low levels of sTWEAK, unlike other cytokines, appear to protect against pathological conditions associated with increased chronic inflammatory activity is incompletely understood. Several conceivable explanations have been proposed. For instance, sTWEAK may have a beneficial effect on the regulation of the immune response because it has been shown that TWEAK-deficiency in mice leads to overabundant natural killer cells and hypersensitivity to bacterial endotoxin, with an excess of interferon-γ and IL-12 production from innate immune cells. Furthermore, sTWEAK inhibits the activation of the transcriptional stimulator signal transducer and activator of transcription (STAT)-1, and induces the association of p65 NF-κB with histone deacetylase 1, thus repressing cytokine production [29]. A reduction of sTWEAK in serum, due to uptake by the Fn14 receptor, has also been postulated. Endothelial dysfunction is the initial pathophysiological step in the progression of vascular damage that precedes and leads to clinically visible CVD [30]. Under these conditions, Fn14 expression is increased in the endothelium. Recently, we reported increased Fn14 expression in human adipocytes from severely obese subjects [31]. These cells also showed an increase in Fn14 expression after inflammatory stimulation, thus increasing availability for the sTWEAK ligand, which could lead to a peripheral reduction in serum sTWEAK [26,27]. An alternative hypothesis proposes the involvement of CD163, a monocyte-macrophage surface receptor which seems to act as a scavenger receptor for sTWEAK [32]. Thus, the reduction in sTWEAK could be related to the presence of sCD163, which is up-regulated in patients with CKD and also in obese subjects [33-35]. This incremental increase could enable sTWEAK degradation by inflammatory macrophages, leading to decreased sTWEAK levels and the reduction in the sTWEAK/sCD163 ratio observed in such diseases as CKD [35]. Thus, low sTWEAK levels may be related to the degree of macrophage activation.

In our study, we found that reduced levels of sTWEAK were not only related to abdominal obesity, but also to such other MetS components as hyperglycemia and hypertriglyceridemia. In this regard, we also found that in human studies, changes in circulating free fatty acids can influence circulating levels of sTWEAK, indicating that lipotoxicity could be an important factor that regulates sTWEAK levels in an inverse manner [27]. Our findings with regard to hyperglycemia are in line with previous studies reporting an inverse association between sTWEAK and such glucose metabolism-related parameters as fasting glucose and HOMA-IR [5,10,15,16]. In fact, impaired fasting plasma glucose levels are recognized risk factors for the development of T2D [36]. Interestingly, in a large prospective nested case–control study we found that reduced sTWEAK levels were associated with T2D incidence [16]. Consistent with our findings, in a study of 60 individuals with chronic hemodialysis (32 had T2D) and 60 controls (30 had T2D), the sTWEAK concentrations in T2D patients on hemodialysis were lower [10]. Conversely, in vitro studies showed that NF-κB activation affects TWEAK-induced insulin resistance by attenuating the action of insulin in hepatocytes [37]. Anyhow, in vivo experiments or animal models may help to clarify the effect of TWEAK on insulin resistance.

At the present time, how TWEAK binding to Fn14 can have such diverse cell-type specific biological effects is not understood, but the differential activation of intracellular signalling cascades is likely to be one explanation. TWEAK can have either positive (beneficial) or negative (detrimental) biological effects, depending on the type of cell, tissue injury and/or the stage of the disease [3,4].

Thus, considering these observations as a whole, we suggest that TWEAK contributes to the inflammatory/anti-inflammatory imbalance observed in obesity, particularly abdominal-visceral obesity and the insulin resistance state, and largely explains the association of TWEAK with hyperglycemia [38] and the future development of T2D. The examination of the biological mechanisms through which sTWEAK improves insulin sensitivity has demonstrated that, in visceral adipocytes, treatment with sTWEAK ameliorates TNFα-induced insulin resistance on glucose uptake [39,40]. This occurs because it abolishes the stimulatory effect of TNFα on JNK1/2 kinase, which is directly involved in the development of insulin resistance [41]. This effect is at least partly produced by a reduction in the cellular concentration of TRAF2, leading to a curbing of TNFα intracellular signaling events. Furthermore, this modulation of TNFα-induced changes in insulin sensitivity was found to be associated with an increase in the activity of PP2A, a Ser/Thr phosphatase, known to negatively regulate cytokine signaling [39]. Additionally, in human subcutaneous adipocytes, sTWEAK exerts a modulatory effect on TNFα-induced cytokine production by inhibiting the MAPK and NF-κB signalling cascades commonly used by TNFα [27].

This protective/modulatory effect of sTWEAK on TNFα activity has been observed in such pathologies as rheumatoid arthritis [42], ischemic stroke [43] and several tumour epithelial cell lines [44], suggesting general competitive behaviour between sTWEAK and TNFα.

Lower levels of sTWEAK have been reported in young individuals with primary hypertension [11]. However, our study conducted in an older population did not provide any evidence to support that reduced levels of sTWEAK are associated with hypertension. The fact that the majority of subjects (90%) were hypertensive might partly explain the lack of associations observed between sTWEAK and the hypertension component of the MetS.

Notwithstanding the results observed, we should point out that our study has several limitations that should be kept in mind. Firstly, a major limitation is that the population studied consisted of elderly Mediterranean Caucasian individuals at high risk for CVD, so our findings cannot be extrapolated to other age groups or ethnicities. Secondly, although rare, we could not completely discard the presence of coronary heart disease in our population explaining the associations observed, given that neither echocardiography nor stress testing were systematically performed in our cohort. Finally, another limitation of our study is inherent to the study design. Because of the cross-sectional nature, conclusions on a cause/effect relationship cannot be drawn.

In conclusion, in the present study we have demonstrated that low TWEAK concentrations are associated with increased risk of MetS, and this relationship was mainly mediated by abdominal obesity, hyperglycemia and hypertriglyceridemia. These findings suggest that sTWEAK may have a key role in the development of these metabolic disturbances, supporting the concept that sTWEAK may be considered as a potential novel biomarker of MetS with a putative protective effect. Further longitudinal studies are warranted to confirm these results in different populations.

## Abbreviations

TWEAK: Soluble TNF-like weak inducer of apoptosis; mTWEAK: Full length TWEAK isoform; sTWEAK: Soluble TWEAK isoform; Fn14: Fibroblast growth factor-inducible 14; PAD: Peripheral artery disease; HOMA-IR: Homeostasis model assessment of insulin resistance; T1D: Type 1 diabetes; T2D: Type 2 diabetes; MetS: Metabolic syndrome; CHD: Coronary heart disease; CVD: Cardiovascular disease; BMI: Body mass index; LDL: Low-density lipoprotein; HDL: High-density lipoprotein; IDF: International Diabetes Federation; AHA/NHLBI: American Heart Association/National Heart, Lung, and Blood Institute; METs: Metabolic Equivalents; NF-κB: Nuclear factor kappa B.

## Competing interests

The authors have no conflict of interest affecting the conduct or reporting of the work submitted.

## Authors’ contributions

AD-L and JS-S had full access to all the data in the study and take full responsibility for the integrity and accuracy of the data analysis. *Study concept and design:* MAM-G, RE, and JS-S *Analysis and interpretation of data*: AD-L, MB, MRCH, JV, MAM-G, and JS-S *Drafting of the manuscript*: AD-L, MRCH, and JS-S *Statistical analysis*: AD-L, MB, and JS-S *Critical revision of the manuscript for important intellectual content*: All authors critically revised and approved the final version of the manuscript.
